# An Implementation Pilot of Web-Based Self-Training Programs on Sexual Dysfunctions in the Dutch Public Sexual Health Setting: Mixed Methods Study

**DOI:** 10.2196/49009

**Published:** 2023-10-26

**Authors:** Filippo Zimbile, Titia Beek, Silke David, Rik Crutzen

**Affiliations:** 1 National Institute for Public Health and the Environment Bilthoven Netherlands; 2 Aidsfonds-Soa Aids Nederland Amsterdam Netherlands; 3 Department of Health Promotion Care and Public Health Research Institute Maastricht University Maastricht Netherlands; 4 Rutgers Expertise Centre on Sexuality Utrecht Netherlands

**Keywords:** implementation pilot digital health, e-sexual health, sexual dysfunctions, self-care, public health, sexual issue, barrier, sexual health, intervention, young adult, young, sexual problem

## Abstract

**Background:**

Web-based sexual health interventions may be more acceptable to people compared with face-to-face support, given the stigma and embarrassment often associated with sexual problems. The Dutch public sexual health clinics (SHCs) conducted an implementation pilot with 4 web-based self-training programs on sexual dysfunctions (WSTPs) for young people. In addition to a basic sexuality program, the WSTPs focused on the following complaints: pain during intercourse, premature ejaculation, and no sex drive.

**Objective:**

This study aims to gain insight into the potential reach of the freely offered WSTPs; use, acceptance, evaluation, and perceived impact of the WSTPs by young people; and evaluation and acceptance of the WSTPs by nurses of the SHCs.

**Methods:**

A quantitative baseline measurement (BM) and a follow-up measurement (FM) were conducted among the users. In addition, qualitative data were gathered through video interviews with a sample of respondents of the FM and nurses of the SHCs to gain more in-depth insights into their assessment of the WSTPs. Participants were recruited via social media, posters, and referrals by nurses of the SHCs. Quantitative data were analyzed using descriptive statistics. Independent 2-tailed *t* tests and one-way independent ANOVAs were used to compare the scores between subgroups based on background characteristics. Dependent 2-tailed *t* tests were used to assess the possible changes between BM and FM. The interviews were analyzed using a thematic analysis.

**Results:**

A total of 1028 young people (aged 16-24 y) completed the BM, 666 started with 1 of the WSTPs, and 104 participants completed the FM. In addition, 8 users and 8 nurses were interviewed. Of the participants who completed the BM, 87.74% (902/1028) experienced moderate (411/1028, 39.98%) to high (491/1028, 47.76%) severity of complaints, of which 20.43% (210/1028) had had them for >1 year and 27.82% (286/1028) even for ≥2 years, and 38.91% (400/1028) were dissatisfied with their sex lives. Only 8.75% (90/1028) had sought professional help in the past 2 years. At FM, users rated satisfaction with their sex life more positively than they did at BM, and they experienced less discomfort from their complaints. The overall rating was positive, with a mean report grade of 7.3 (SD 1.45; on a 10-point scale). Anonymity, clear information and explanation, and practical exercises are indicated as strengths of the WSTPs, leading to more understanding and normalization. Nurses appreciate the high quality of information and accessibility of the WSTPs. They consider them as a valuable addition to the consultation hours.

**Conclusions:**

WSTPs can reach a large number of young people with sexual problems who are less likely to seek professional help. This can result in an improved understanding of their issues, a decrease in complaints, and reduced barriers to communicating with a partner or professional.

## Introduction

### Background

Sexual dysfunctions and difficulties concern a wide range of problems related to sexual desire and interest, arousal, orgasm, and sexual pain [1]. These problems can negatively influence the quality of life and lead to decreased relationship satisfaction [2-4]. Among sexually active respondents (aged 40-80 y) in an international study, 43% of men and 49% of women experience at least 1 sexual problem during the last 12 months [5]. A recent estimation of the prevalence of sexual dysfunctions in Germany reported ≥1 sexual problems in the previous 12 months in 33.4% of men and 45.7% of women [6]. A study in the Netherlands among sexually active young people (aged 12-24 y) showed that premature ejaculation is the most frequent problem among young men: 25% experienced this problem in the last 3 months, and 5% experienced this problem often and indicated that it is (quite or very) bothersome. Among young women, problems related to orgasm (39%), no sex drive (34%), lubrication problems (31%), and pain during intercourse (27%) are frequently occurring problems [7]. The results of this Dutch study are consistent with those of young people in other countries [8].

Despite the prevalence of these sexual problems, there are both individual-level and system-level barriers to accessing help from a health professional [9]. These barriers include not knowing where to seek help, a lack of specialty services, waiting lists, high costs for private services, and discomfort talking about sex with professionals [10-12]. Even within the group of young people indicating that frequent and recurring sexual problems bother them quite or very much, about two-thirds do not seek help. Only 8% of boys and 11% of girls go to a counselor or physician [7]. When young people seek help, almost 1 in 8 young people seek help on the web or from friends, acquaintances, or family.

To improve sexual health among young people, the 24 public sexual health clinics (SHCs) in the Netherlands offer free, anonymous consultations (ie, Sense consultations) on a broad range of subjects related to sexual health, including (problems with) sexual intercourse, unwanted pregnancy, birth control, sexually transmitted infections (STIs), sexual orientation, sexual violence, and exploitation. In 2021, the total number of consultations at the SHCs was 138,436 [13]. As in previous years, the vast majority of consultations were related to STI testing. Approximately 10% dealt with other questions about sexual health issues: 8410, 3224, and 321 Sense consultations were registered in 2021 among women, men, and transgender or intersex persons, respectively.

As the SHCs want to reach more susceptible young people and more men, they are interested in the opportunities offered by web-based services for sexual health. In the international literature, there are several examples of how this could work well, and a number of relevant benefits emerge: because young people are almost constantly on the web, digital services provide a way of reaching them that is not threatening, familiar, and intuitive [14]. Furthermore, web-based interventions offer the possibility to individually tailor intervention content, potentially leading to more interactivity, customization, and engagement by the user [14]. As a result, web-based interventions can be a cost-effective alternative to face-to-face help [14,15]. Finally, the privacy and anonymity of web-based interventions is an important benefit, especially given the stigma and embarrassment often associated with experiencing sexual difficulties [16]. Because of the reduced embarrassment and shame, web-based sexual health interventions may be more acceptable to people compared with face-to-face support and will lead to increased understanding of their sexual difficulties, increased sexual confidence, and a better feeling of being able to speak to a health professional about the difficulties [9]. However, in general, patients are more enthusiastic about web-based technology than medical professionals. Concerns of the professionals are related to the perceived quality of diagnosis and interaction with patients [17].

### Objectives

Together with the Dutch national expertise centers on sexual health Soa Aids Nederland (focus on HIV and STIs), Rutgers (focus on sexual and reproductive health and rights), and the National Institute for Public Health and the Environment (*Rijksinstituut voor Volksgezondheid en Milieu*), the SHCs started in 2022 an implementation pilot offering web-based self-training programs on sexual dysfunctions (WSTPs), branded as the Sense Online Coach (SOC). Sense is the Dutch brand name of the public sexual health services for young people. Sense services are offered by the previously mentioned cooperating organizations. The objective was to investigate the extent to which WSTPs are acceptable and attractive for young people with sexual problems and to reach people who are less likely to seek face-to-face help from a health professional at a clinic.

The objective of the implementation pilot was to gain insight into (1) the potential reach of the free WSTPs; (2) use, acceptance, evaluation, and perceived efficacy of the WSTPs by young people; and (3) assessment and evaluation of the WSTPs by nurses of the SHCs.

This study does not focus on the evaluation and effectiveness of each WSTP specifically but on general features that can be relevant to the developers of similar services.

## Methods

### Overview

A mixed methods approach was used to achieve the objectives of the pilot. In Table 1, we have operationalized the objectives of the pilot into research topics.

Among the WSTP users, a quantitative baseline measurement (BM) and a follow-up measurement (FM) were conducted as part of routine care. In addition, qualitative data were gathered through video interviews among a sample of respondents of the FM to obtain more detailed insight to facilitate the interpretation of the quantitative data. Nurses of the SHCs were invited to participate in video interviews to evaluate the WSTP.

In the pilot, four different types of WSTPs were offered:

1. A basic sexuality program (participants who were interested in the other WSTPs were advised to start with this WSTP, but it can also be used as a stand-alone program)
2. A program aimed at reducing pain during intercourse (for women)
3. A program aimed at reducing premature ejaculation or orgasm (for men)
4. A program aimed at supporting persons who are bothered by a lack of or limited sex drive.

**Table 1:** Research questions of the pilot study.

Objective and topics		BM^a^	FM^b^	Interviews
**Potential reach**				
	Route to the WSTP^c^	Social media Local adds Consultation hours	N/A^d^	Topic included
	Demographic characteristics of young people reached	Age Sex Educational level Sexual orientation	N/A	Topic included
	Background on sexual help requests from people reached	Satisfaction with sex life Severity of the complaints Duration of the complaints Help-seeking behavior in the past 2 y	N/A	Motivation to participate and expectations before the start, choice of program or programs
Use of the WSTPs		N/A	Completion of the WSTP Per program Reasons for dropout	Completion of the programs and reasons for dropout (when applicable)
Acceptance and evaluation of the WSTPs		N/A	Overall rating Trustworthiness information Videos and assignments Degree of incitement to action Usefulness for peers with similar problems Strengths and weaknesses of WSTPs	Evaluation of the WSTPs (perceived strengths and weaknesses)
Perceived impact and efficacy		Observed differences between scores in BM and FM on: Satisfaction with sex life Severity of the complaints	Observed differences between scores in BM and FM on: Satisfaction with sex life Severity of the complaints	Perceived impact and efficacy of the WSTPs

^a^BM: baseline measurement.

^b^FM: follow-up measurement.

^c^WSTP: web-based self-training programs on sexual dysfunction.

^d^N/A: not applicable.

### Background and Explanation of the WSTPs Used

The SOC was conducted in collaboration with a Dutch commercial provider of WSTPs on various (mental) health topics (ie, Gezondeboel.nl). The reason for seeking cooperation with this company was that they offer user-friendly programs with video coaches, visuals, and animations tailored to a younger target group. Three existing programs of Gezondeboel.nl were selected for the pilot (basic program, pain during intercourse, and [no] sex drive), and a new program was developed in collaboration with the Sense partners (premature ejaculation). All programs were developed by sexologists associated with the Dutch Society of Sexology (*Nederlandse Wetenschappelijke Vereniging voor Seksuologie*) in alignment with the standards for sexual problems of the Dutch General Practitioners Association (*Nederlands Huisartsen Genootschap*). The existing programs were first reviewed by 2 sexologists from 2 different SHCs and adjusted slightly to meet the guidelines of the SHC. Owing to limited possibilities to gather data within the programs and very limited response from the commercial users, only limited evaluation data were available on the sexual health programs before the start of the pilot.

The 4 WSTPs have the same format and structure. The user can select a video coach that appeals most (based on age and sex). The video coach explains the program and its different sessions and guides the participant through the program. The sessions consist of information, examples, questionnaires, and different types of assignments ranging from compiling mood boards, drawing, writing film scripts to physical exercises. Participants can perform an assignment on their own but, in most cases, also with a partner.

The programs focused on awareness and patterns of behavior, followed by changes in behavior. All 4 programs were explicit psychological interventions that used aspects of cognitive behavioral therapy and positive psychology. As most participants have sex with a partner, the principles of system therapy were also applied. Although evidence varies, (web-based) psychological interventions have proven to be effective treatment options for sexual dysfunctions [18-20].

The WSTPs are described in more detail in Table 2.

As all WSTPs are exclusively psychological interventions, attention is paid at the beginning of each program to signals that may indicate physical or medical complaints. Participants who recognize themselves in this are advised to also consult their general practitioner. Furthermore, each program is concluded with the remark that it is very well possible that the program has not helped or has not helped sufficiently. For those who recognize themselves in this, an overview has been included with possible care providers (such as sexologists, health information specialists at the Sense helpline, and general practitioners) and an explanation of how to get in touch with these professionals. Furthermore, the following statement is included: “Don’t hesitate! Professional care providers are there to help you on your way.”

**Table 2:** Description of the content of the web-based self-training programs on sexual dysfunctions (WSTPs).

WSTP	Content	Target group
Basic sexuality program	Introduction What is sex? Your sex How does sex work? Sex in different stages of life: physical and medical complaints Communication about sex (assignments) Quantum model (assignment) You have to learn sex Dealing with discomfort Your wishes	Boys and girls
Pain during intercourse	Introduction What is pain during intercourse? Why do I have pain? Should I first visit a general practitioner? (complaints that indicate possible medical or physical causes of the problem) Assessment current situation (levels of pain, arousal, and relaxation) Conditions of sex without pain Pelvic floor, arousal, explanation of the pain circle (assignments) Look and feel Exercises: how does my vulva look Exercises: inserting something into your vagina Sexual arousal (assignments) Self-image, looking for erotic stimuli, concentration exercises, and using your fantasy Involving your partner and having sex together (assignments)	Girls
Premature ejaculation	Introduction What is an orgasm or ejaculation? What is premature ejaculation (assignment)? Myths (assignments) Causes Mental, physical, and environmental (assignments) What can you do? Preparation Exercise: lower arousal Exercise: use lubricant Exercise: squeeze technique (masturbation; assignment) Pelvic floor exercises Communication with your partner Variation in sex positions	Boys
Lack of or limited sex drive	Introduction Conditions for pleasurable sex Circle of negative and positive expectations Common misunderstandings about sex Reasons to have sex Reasons for men and women to have no sex drive Documentary: “my sex is broken” Assignments How to get in the mood for sex What do you need? How can you get in the mood for sex (assignment)? Being curious about your partner (assignment) Attention to sexual stimuli (assignment) Sex is something you need to do Sexual desires without words (assignment) Doing what you like (assignment) Exploration of arousal enhancing clothing, toys, and tools Longing for each other (assignment)	Boys and girls

### Study Design

**Figure 1:**
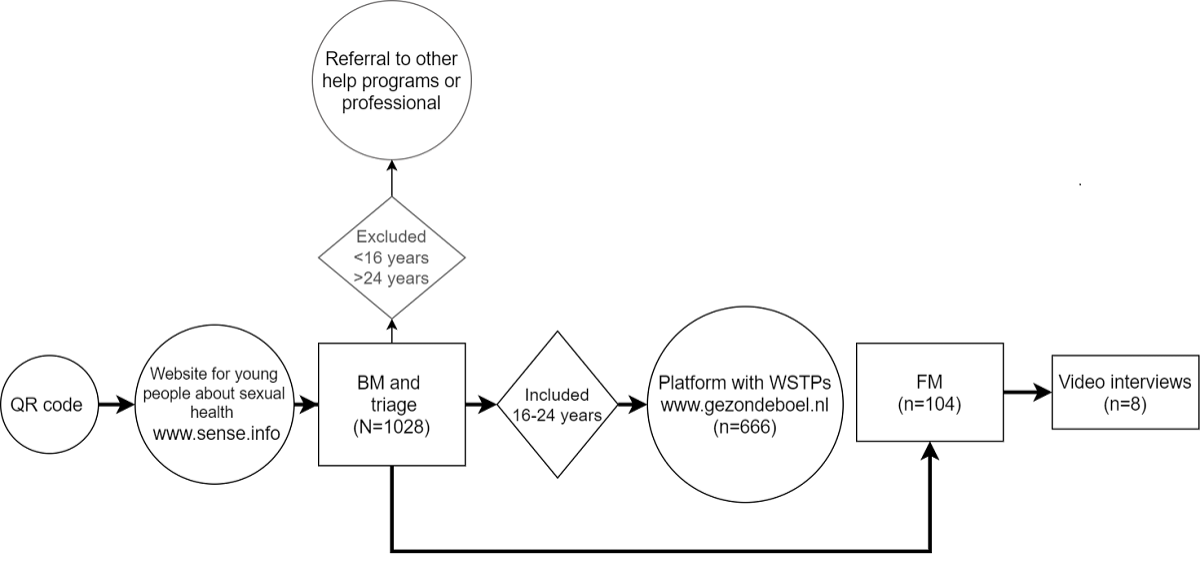
Flowchart of the pilot implementation of the Sense Online Coach mixed method study. BM: baseline measurement; FM: follow-up measurement; WSTP: web-based self-training programs on sexual dysfunction.

This study was performed among young people (aged 16-24 y) in the Netherlands. Youth aged <16 years were excluded because parental consent was required for this target group, and this was not deemed appropriate given the sensitivity of topics in the SOC and their private nature. Individuals >24 years were also excluded because they were not eligible for the free public services of the SHCs. They were referred to corresponding commercial web-based training courses. Quantitative data collection was conducted between April 4 and October 31, 2022. Figure 1 presents the graphical study flow of the pilot.

Participants for the SOC have been recruited in various ways by the SHCs:

1. On the national level, by social media advertisements directed at the target groups performed by a specialist social media agency
2. On the local and regional level, by the SHCs themselves by distributing posters or handing out cards (with QR codes to additional information and the web registration page) at locations such as waiting rooms at the clinic, schools, and community centers where the target group is regularly present
3. On the consultation hour level at the SHC, by a nurse who refers a client in response to the complaints that have arisen during a consultation (eg, an STI consultation). In consultation between a nurse and client, an appointment for a follow-up consultation could also be made to discuss the experiences and results of the WSTP and possible follow-up steps.

### Protocol and Participants

Potential participants landed on a specially designed Sense landing page. This landing page explained what the SOC is and how it works. It was also explained that it concerned a pilot project and that only young people who wanted to participate in the research could participate. Those interested but who did not want to participate in the study or who fell outside the age category of the target group were referred to the commercial offer (€49 [US $51] per program).

Only people who had fully completed the BM were referred to the free-of-charge WSTPs and were immediately given the opportunity to create a personal account and start with the selected program. Three weeks after completing the BM, all participants received an invitation by email to complete the FM. An incentive worth €7.50 (US $7.72; gift card) was offered to encourage as many people as possible to participate in the FM. A reminder was sent again at a later time.

At the end of the FM questionnaire, participants were invited to sign up for additional telephone interviews to evaluate the care provided to them. If they were willing to do so, an incentive of €10 (US $10.43; gift card) was offered. Nurses who referred clients to the SOC or planned a follow-up consultation were requested to motivate them to complete the questionnaire and to sign up for the interviews.

Most nurses from the public SHCs who perform the consultations have completed additional training in the field of sexual health. In the context of prevention and health support, they offer young people (aged 12-24 y) sex counseling. This consists of a limited number of consultations for young people who have problems with sex and sexuality. These nurses were trained to recognize the signs of possible sexual problems. During the pilot period, they were given the opportunity to refer young people to the WSTPs if they judged that it would fit the needs of the young person. For this purpose, they had business cards available with a QR code that led to the SOC landing page. During the pilot study, nurses were advised that they could offer the WSTPs in combination with a follow-up consultation. Before the start of the pilot study, nurses from all SHCs received information and instructions about the WSTPs and how young people could be referred. They were also given the opportunity to become acquainted with the 4 programs offered through a trial account. Questions and comments could be shared with regional eHealth coordinators who were involved in setting up the pilot. At the end of the pilot period, nurses were invited by the regional coordinators to participate in the evaluation of the pilot, and they were also offered an incentive of €10 (US $10.43; gift card).

### Ethical Considerations

#### Human Participant Ethics Review Approvals or Exemptions

As this study is an implementation pilot as part of regular care and not a medical scientific research study in the Netherlands, no approval is necessary from a medical ethical committee [21]. Despite this, we paid a lot of attention to ensuring that participants and their data were handled in a safe and careful way in the pilot. We describe this in more detail for each of the study activities in the following sections.

#### Informed Consent, Privacy, Confidentiality, and Compensation Details

##### BM and FM

All youth interested in participating in ≥1 WSTPs were directed via the promotional materials to a landing page on the national public sexual health website Sense.info. On this page, they were informed about the pilot and that free use of the WSTPs was possible. Those willing to participate in the pilot study were informed that their data were being processed, and a link to the privacy statement was provided before they were referred to the BM. The privacy statement described which data were used for what purpose and which data were shared with other organizations (eg, Rutgers and the provider of the WSTPs).

In the introduction of the BM, it was explained that all data were handled with care: personal data were deleted as soon as they were no longer required for analysis. It was also indicated that name, email address, or other data that could be traced back to the participants were not included in the reports. Participants were reminded that they did not have to use their real name but that they always had to use the same email address. Furthermore, the FM was announced that they would receive in 3 or 4 weeks on their email address. It was explained that participation in the FM was not mandatory. It was indicated that participants in the FM would receive a gift card worth €7.50 (US $7.72). This information was followed by the participants who opted to register for the SOC.

At the end of the FM, respondents were asked whether they would like to participate in telephone interviews. Those who were prepared to do so were offered an incentive of €10 (US $10.43).

##### Interviews Participants and Nurses

At the beginning of the interviews, all interviewees and nurses were informed that participation was voluntary and that they could stop at any time during the interview. They were asked for permission to make audio recordings of the conversations and explained that their data would only be used for the pilot and that the data would be kept confidential. Before the start of each interview, all the participants were asked to provide explicit permission. The nurses also received an incentive of €10 (US $10.43) for participating in the interviews.

### Data Collection

#### BM Participants

The 12-items web-based questionnaire for the BM consisted of 3 parts. The first part contained information about the background, objective, and design of the pilot. Here, the FM by email was also announced. Participants were informed that SOC participation was under the condition of completing the BM. They were asked to always use the same email address and were allowed to use a nickname. Those who did not consent to the abovementioned criteria or were out of the age range were referred to a commercial provider. The second part of the questionnaire contained questions about the participants’ background, such as sex, sexual preference, educational level, place of residence, how they found the SOC, and email address. The third part of the questionnaire addressed satisfaction with their sex life (5-point Likert scale ranging from very satisfied to very dissatisfied), duration of the complaints (6-point Likert scale ranging from <1 mo to ≥2 y), a rating about the burden they were experiencing (ranging from 0=no burden at all to 10=extremely burdened), and whether they had received help for their problems in the past 2 years by a professional (yes, no, and do not know). The questionnaire ended by asking participants which program they wanted to follow.

#### FM Participants

The follow-up questionnaire contained 12 items, starting with the status of the WSTP (completed, still in progress, and stopped before completion) and motivation for stopping before completion if that was the case (checkboxes: complaints are reduced before the program ended, I felt I had learned enough, the program made no sense, and different reason with open space to explain). Satisfaction with their sex life and the rating of the experienced burden were measured in the same way as in the BM. In addition, the respondents were asked to indicate (on a 5-point Likert scale ranging from totally disagree to totally agree) whether the program has reduced their complaints, whether the program is also useful for other young people with similar problems, and the usefulness of the informational videos and assignments. The extent to which they trusted the program’s information and felt they were empowered to take action by the program was measured using a 7-point Likert scale (ranging from totally disagree to totally agree). The program as a whole was evaluated using a report grade (1-10) and 2 open-ended questions about what they liked about the program and what opportunities for improvement they saw.

In case a follow-up consultation was planned by a nurse, the respondent was also asked to answer the following item: indication of satisfaction with the follow-up consultation (5-point Likert scale, ranging from very satisfied to very dissatisfied).

#### Qualitative Interviews Participants

The participants who had indicated (in the FM) willingness to participate in the follow-up qualitative evaluation were approached by the interviewer. The interviewer conducted individual interviews by video, which lasted approximately 15 to 30 minutes. In the interviews, participants were asked for consent to participate in the study and were informed that participation was anonymous. A semistructured interview guide containing 18 open-ended questions relating to the assessment of the program or programs and factors contributing to this assessment was used. The following main topics were addressed: demographic characteristics, route to the WSTP, motivation to participate and expectations before the start, choice of program or programs, completion of the programs and reasons for dropping out (if applicable), perceived impact and evaluation of the OSTP (perceived strengths and weaknesses), and evaluation of follow-up consultation (if applicable).

#### Qualitative Interviews Nurses

For the video interviews with the nurses, a semistructured interview guide was used, containing five major themes: (1) current use of the WSTPs and underlying motivation, (2) whether or not follow-up consultations were offered and the perceived added value of the WSTPs this consultation, (3) satisfaction with the current WSTPs, (4) the method of implementation, and (5) the way in which the WSTPs could be used in the future and need for additional WSTPs. The interviews lasted approximately 45 minutes and were conducted by the same interviewer who conducted the interviews with the participants.

The web-based questionnaires and video interview guides for participants and nurses and the codes generated during the analyses of the qualitative data are available as web-based appendixes at Open Science Framework SOC [22].

### Data Processing and Analyses

#### Web-Based Questionnaires

SPSS Statistics (version 27.0; IBM Corp) was used to provide descriptive statistics for the items of the quantitative BM and FM. To establish differences between groups based on age category, educational level, and sex (because of the underrepresentation of the category “other,” only men and women were compared in the analysis) on the variables “satisfaction with sex life,” “duration of the complaints,” and “perceived severity of the complaints,” chi-square tests for categorical variables and independent 2-tailed *t* tests for continuous variables (if the independent variable had 2 categories) or one-way ANOVAs (if the independent variable had >2 categories) were used. Robust Welch F (including adjusted *df*) were reported when groups had unequal variances. Regular *F* values were reported when the groups had equal variances.

To assess the potential reach and impact of the SOC from the baseline and FMs a dependent 2-tailed *t* test was used to assess a possible change between the baseline and FM for the variables “satisfaction with sex live” and “perceived severity of the complaints.” To assess whether differences in perceived satisfaction and severity of complaints (gain scores) are related to sex, age, or educational level, one-way independent ANOVAs and independent 2-tailed *t* tests were used.

#### Video Interviews

All completed interviews were transcribed, and the names of the participants were deleted. MAXQDA2022 (Verbi Software GmbH) was used to code and analyze the qualitative data. A thematic analysis was used. The themes were deductively generated based on general prespecified assessment criteria: expectations before starting, motivations for subscribing, reasons (not) to complete the program, advantages and disadvantages of WSTP, perceived impact, and recommendations for future implementation. At the same time, the possibility was left open to inductively add codes based on data.

## Results

### Baseline Measurement

A total of 1248 individuals completed the BM. After removing test accounts (4/1248, 0.32%), persons who did not want to participate in the pilot (121/1244, 9.73%), persons aged ≤15 years (20/1123, 1.78%) or ≥25 years (63/1123, 5.61%), and duplicates, the data of 1028 participants were included in the analyses.

### Follow-Up Measurement

Anonymous user statistics from the commercial provider of the SOC show that not all young people who completed the BM created an account. A total of 855 (83.17% of valid BM) accounts out of 1028 were created. A total of 774 programs were started by 666 (64.8% of valid subscribers) clients, 333 (50%) clients completed at least 50% of the program, and 126 (19.4%) clients completed >80%.

A total of 190 individuals used the link to the FM. After removing duplicates and errors regarding email addresses, 104 respondents (15.6% of all subscribers who actually created an account) were included in the analyses of follow-up data.

### Missing Data Handling

In general, data were not imputed when missing values were present. Participants were excluded from certain (descriptive) analyses if they had missing values for a specific variable. More specifically, for the comparison between the pre- and postmeasurement for each of the outcome variables, only participants with valid data on both the baseline and FM were included. When data were missing on either of the measurements, the participant was excluded from that analysis.

### Video Interviews: Young People

A total of 8 young people participated in the interviews: 6 female participants and 2 male participants. Most of them had a higher level of education (6/8, 75%) and 2 (25%) had an intermediate level. Except for 1 (13%) woman (bisexual), all of them (7/8, 88%) were heterosexual in the age group of 20 to 29 years.

### Video Interviews: Nurses

From November 2022 to January 2023, a total of 8 nurses who had referred clients during consultation hours to the SOC from 6 different SHCs were interviewed.

### Results of the BM

#### Route to the SOC and Selection of the Programs

A large majority (841/1028, 81.81%) of the young people found the SOC via social media advertisements. Relatively few young people entered the SOC via the other channels: 5.35% (55/1028) through posters or cards at schools, community centers, or at the SHC. Only 2.72% (28/1028) were referred by a nurse. The other 10.12% (104/1028) found the SOC in a different way (friends, search on the internet, or via other health professionals). Of the participants reaching the SOC via social media (841/1028, 81.81%), 46.1% (388/841) experienced a high level of complaints and 41.5% (349/841) experienced a medium level. Of the participants reaching the SOC via a nurse (28/1028, 2.72%), 36% (10/28) experienced a high level of complaints and 46% (13/28) experienced a medium level. A comparison between these channels with regard to perceived satisfaction with their sex life shows that 36.6% (308/841) of the subscribers via social media are (very) dissatisfied with their sex life and 39.7% (334/841) are neutral; for those who subscribe via a nurse, these percentages are 25% (7/28) and 50% (14/28), respectively.

The most selected program was “premature ejaculation” (414/1028, 40.27%, of which 407/414, 98.3% was male), followed by “(no) sex drive” (362/1028, 35.21%, of which 288/362, 79.6% was female) and “pain during intercourse” (207/1028, 20.14%, of which 190/207, 91.8% was female). The basic program as stand-alone program was selected by 4.38% (45/1028), of which 62% (28/45) was male.

#### Demographic Characteristics

The demographic characteristics and background of the young people who completed the BM and got access to the SOC are summarized in Table 3.

The selection of programs by sex is presented in Table 4. The SOC appeared to be equally interesting for both men (525/1028, 51.07%) and women (497/1028, 48.35%).

The youngest category (16-18 y; 117/1028, 11.38%) was underrepresented compared with the category of 19-21 years (339/1028, 32.98%) and 22-24 years (511/1028, 49.71%). The majority (618/1028, 60.12%) had a higher educational level, 34.92% (359/1028) had an intermediate level, and only 4.86% (50/1028) had a lower level. A vast majority (835/1028, 81.23%) were heterosexual, 10.8% (111/1028) were bisexual, and 5.45% (56/1028) were homosexual or lesbian.

**Table 3:** Demographic characteristics and background of the participants at baseline (N=1028).

Characteristics			Participants, n (%)
**Age group (y; n=1027)**			
	16-18	117 (11.39)	
	19-21	399 (38.85)	
	22-24	511 (49.76)	
**Educational level (n=1027)**			
	Low	50 (4.87)	
	Intermediate	359 (34.96)	
	High	618 (60.18)	
**Sex (n=1027)**			
	Female	497 (48.39)	
	Male	524 (51.02)	
	Intersex	3 (0.29)	
	Transgender	1 (0.1)	
	Not given (other)	2 (0.19)	
**Sexual orientation (n=1027)**			
	Heterosexual	835 (81.3)	
	Homosexual	56 (5.45)	
	Bisexual	111 (10.81)	
	Other	6 (0.58)	
	Do not know	19 (1.85)	
**Severity of complaints**			
	High (7-10)	491 (47.76)	
	Moderate (4-6)	411 (39.98)	
	Low (0-3)	126 (12.26)	
**Sought professional help in the last 2 y**			
	Yes	90 (8.75)	
	No	921 (89.59)	
	Do not know	17 (1.65)	
**Satisfaction with sex life**			
	Very satisfied	30 (2.92)	
	Satisfied	210 (20.43)	
	Neutral	388 (37.74)	
	Dissatisfied	318 (30.93)	
	Very dissatisfied	82 (7.98)	
**Duration of sexual complaints**			
	≤1 mo	71 (6.91)	
	1-3 mo	123 (11.96)	
	3-6 mo	155 (15.08)	
	6-12 mo	183 (17.8)	
	1-2 y	210 (20.43)	
	≥2 y	286 (27.82)	

**Table 4:** Selection of program by sex.

Choice of program	Male (n=525), n (%)	Female (n=497), n (%)	Intersex, transgender, and not given (other; n=6), n (%)	Total (N=1028), n (%)
Basic program	28 (5.3)	17 (3.4)	0 (0)	45 (4.4)
(Basic) and premature ejaculation	407 (77.5)	5 (1)	2 (33.3)	414 (40.3)
(Basic) and (no) sex drive	71 (13.5)	288 (57.9)	3 (50)	362 (35.2)
(Basic) and pain during intercourse	19 (3.6)	187 (37.6)	1 (16.7)	207 (20.1)

#### Backgrounds of Help-Seeking Behavior: The Extent to Which WSTPs Reach Young Persons With Sexual Dysfunctions?

Of the 1028 young people who completed the BM, the average score for satisfaction with one’s own sex life was 3.2 (SD 1.0) on a 5-point Likert scale (5=very dissatisfied). Overall, 38.91% (400/1028) of the subscribers were (very) dissatisfied, one-third neutral (388/1028, 37.74%), and approximately a quarter (very) satisfied (240/1028, 23.35%). The participants in the youngest age category (16-18 y) were more satisfied than those in the oldest category (22-24 y; *F*_2;324,9_=3.1; *P*=.04), and men (mean 3.0, SD 1.0) were more likely to be satisfied with their sex life than women (mean 3.43, SD 0.9; t_1020_=−7.58; *P*<.001).

These results are in line with the participants’ own assessment of the severity of their complaints: women experienced a higher level of complaints (mean 6.4, SD 2.0) compared with men (mean 5.9, SD 2.2; t_1020_=−3.98; *P*<.001). Participants in the youngest age category (16-18 y) experienced a lower level of complaints than those in the oldest category (22-24 y; *F*_2;309,7_=4.2; *P*=.02).

Almost half of the subscribers (496/1028, 48.25%) reported experiencing sexual problems for ≥1 year. For approximately one-fifth (194/1028, 18.87%) of the subscribers, this period was ≤3 months. A large majority (921/1028, 89.59%) did not seek any professional help in the past 2 years for their sexual problems. As shown in Tables 5 and 6, a large majority of the subscribers with a high self-reported level of complaints (421/491, 85.7%) had not sought professional help in the last 2 years. In the group with a moderate degree of complaints, this percentage was 92.2% (379/411).

More than half (266/491, 54.2%) of the subscribers who experienced a high level of complaints were dealing with these problems for ≥1 year, and about one-third (154/491, 31.4%) were dealing with them for even >2 years. There was a positive association between the duration of sexual complaints and perceived severity of complaints (*χ*^2^_10_=41.8; *P*<.001).

**Table 5:** Received professional help in the past 2 years by self-assessed degree of complaints (N=1028).

Degree of complaints	Received professional help in the past 2 y, n (%)			
	Yes	No	Do not know	Total
Low (0-3)	3 (2.4)	121 (96)	2 (1.6)	126 (12.3)
Moderate (4-6)	25 (6.1)	379 (92.2)	7 (1.7)	411 (40)
High (7-10)	62 (12.6)	421 (85.7)	8 (1.6)	491 (47.7)

**Table 6:** Degree of complaints by received professional help in the past 2 years.

Received professional help in the past 2 y	Degree of complaints, n (%)			
	Low (0-3)	Moderate (4-6)	High (7-10)	Total
Yes	3 (3.3)	25 (27.8)	62 (68.8)	90 (8.8)
No	121 (13.1)	379 (41.2)	421 (45.7)	921 (89.6)
Do not know	2 (11,8)	7 (41.2)	8 (47.0)	17 (1.7)

### Results of the FM: Use, Acceptance, and Evaluation of the SOC by Participants

More respondents with a higher educational level (74/104, 71.2% vs 618/1027, 60.18%), less younger respondents (7/104, 6.7% vs 117/1027, 11.39%), and more women (65/104, 62.5% vs 497/1027, 48.39%) were represented in the FM compared with the BM. An overview of the demographic characteristics of the respondents in the FM is available as a web-based appendix at Open Science Framework SOC.

#### Motivations for Quitting or Drop Out

In Table 7 is shown for each program how many young people in the FM have completed the program.

The user statistics and results of the FM showed that the dropout rate was considerable (Table 7). Subscribers who started with the SOC but did not complete the program had the following motivations: approximately one-third (16/52, 31%) of them indicated that their complaints had reduced. An equal proportion (16/52, 31%) of participants stated that they felt they had learned and practiced enough. A minority (7/52, 13%) explained that they did not find the SOC meaningful. One-third (18/52, 35%) had another reason to stop the training; most mentioned reasons were the ending of a sexual relationship, their sex partner did not like it, lack of time owing to other obligations, their complaints, and the content of the WSTPs did not match (different expectations). Analyses did not show any relationship between the level of complaints experienced and satisfaction with their sex lives in the BM and completion of the SOC measured in the FM.

**Table 7:** Program or programs selected and completion (yes or no) by respondents of the follow-up measurement (N=104)^a^.

Program	Yes, n (%)	No, n (%)	Total, n (%)
Premature ejaculation (n=32)	17 (53)	15 (47)	32 (100)
No sex drive (n=42)	18 (43)	24 (57)	42 (100)
Pain during intercourse (n=27)	12 (44)	15 (56)	27 (100)
Basic training (n=3)	2 (67)	1 (33)	3 (100)

^a^Yes: 49 (47%), no: 55 (53%), total n=104.

#### Acceptance and Evaluation of the SOC

After using the SOC, participants assessed satisfaction with their sex life, on average, significantly more positively (mean 2.6, SD 1.0; t_94_=6.78; *P*<.001; Cohen *d*=0.88) than they did in the BM (mean 3.2, SD 1.0). The participants also reported, on average, experiencing significantly less discomfort from their complaints in the FM (mean 4.76, SD 2.43; t_94_=4.55; *P*<.001; Cohen *d*=0.47) than in the BM (mean 5.83, SD 2.31).

The respondents of the FM were asked to evaluate the different aspects of the SOC. The results are summarized in Table 8.

The videos were evaluated most positive. A large majority of the respondents (83/100, 83%) qualified them as (very) useful. Only a few respondents (4/100, 4%) assessed the video as (very) useless. The assignments were appreciated a bit less compared with the videos; still, a majority found them (very) useful (58/100, 58%), and a limited number of respondents (8/100, 8%) found them (very) useless.

In Table 9 it is shown that a large majority of the respondents (83/100, 83%) thought that the SOC was useful for other young people with comparable sexual complaints. Two-thirds (66/100, 66%) of the respondents trust the information on the SOC, whereas a substantial proportion (20/100, 20%) do not trust it. A small majority of the respondents (52/100, 52%) agreed to some extent that they could take action based on the SOC. About one-third (35/100, 35%) of them disagreed.

The respondents were asked to give the SOC an overall rating. The mean grade was 7.3 (SD 1.45; median 8) on a 10-point scale.

**Table 8:** Evaluation of the different aspects of the Sense Online Coach.

	Videos (n=100), n (%)	Assignments (n=100), n (%)
Very useful	19 (19)	14 (14)
Useful	64 (64)	44 (44)
Neutral	13 (13)	34 (34)
Useless	3 (3)	8 (8)
Very useless	1 (1)	0 (0)

**Table 9:** Assessment of usefulness, information, and activation.

	Useful for others (n=100), n (%)	Trust the information (n=100), n (%)	I can take action (n=100), n (%)
Totally agree	23 (23)	31 (31)	9 (9)
Agree	60 (60)	35 (35)	24 (24)
Slightly agree	N/A^a^	5 (5)	19 (19)
Neutral	14 (14)	4 (4)	13 (13)
Slightly disagree	N/A	8 (8)	19 (19)
Disagree	3 (3)	12 (12)	7 (7)
Totally disagree	0 (0)	15 (15)	9 (9)

^a^N/A: not applicable.

#### Strengths and Weaknesses of the SOC

Respondents were given the opportunity to identify the strengths and weaknesses of the SOC through open questions; 79 respondents have done this. The most mentioned strengths were clear information and explanation (52/79, 66%), useful exercises (14/79, 18%), the SOC being accessible and anonymous (10/79, 13%), variation in the form (9/79, 11%), and the possibility of choosing between different coaches (8/79, 10%).

Weaknesses or points of improvement were mentioned by 62 respondents. The most frequently mentioned points of attention were regarding the content of the programs: more depth of information; more variance and more practical exercises; and more stimulating, interesting, and spontaneous content were mentioned equally often (7/62, 11%). Furthermore, concrete guidelines for the next steps after the SOC and more actively motivating and remembering participants to complete the WSTP were mentioned as improvements (both 6/62, 10%).

### Qualitative Evaluation by Participants

#### Help-Seeking Behavior and Expectations of the SOC

Several participants were not actively seeking help for their sexual health problems. They do not see their situation as a real problem but more as a struggle. Some became curious after seeing an advertisement on social media:

I already said: I didn’t have a real help request...Putting it a bit rudely, but very horny or very wet is difficult for me.

I was just busy online, I think there was an advertisement about no sex drive at the time, and then I thought: yes, you know this applies to me.

Others were looking specifically for help and ended up at the SOC after searching the internet or were referred by a nurse during a consultation for STI testing at an SHC. In 1 case, the SOC attracted attention because a partner was experiencing sexual problems, and the participant was curious if the SOC could help them.

Nearly all the respondents had no expectations of the SOC. They started with an open mind and were curious. The fact that there is no charge for the SOC makes it easy to give it a try:

If I get something out of it, that’s a bonus, otherwise I’ll lose a few hours of my life. That doesn’t hurt.

Those who had clear expectations expected information, advice (tips and hints), and exercises.

#### Reasons for Not Completing the SOC

The participants who had not fully completed the SOC give various reasons for this. Several participants stopped because while following the program, they got the impression that it would not help them any further; for example, the information provided was judged to be insufficiently specific to their personal situation:

Yes, I have stopped before. It’s more the information that was in it, it was kind of the basics. All a bit simple.

Others indicate that they have received too few reminders or incentives from the program to complete it:

Well, there wasn’t really a deadline on it and I kind of forgot.

In addition, personal circumstances also play a role, such as ending a relationship or a partner who no longer wanted to continue with the SOC.

#### Strengths of the SOC

If the SOC is considered in general, not by the separate WSTPs, the following strong elements emerge: clear explanation, exercises, videos in combination with written text (the literal words spoken in the videos), the level and construction of the modules, and the possibility of choosing one’s own coach.

Some of the participants appreciated the library and the referrals to additional information because they needed more in-depth or specific information than was offered in the programs.

All participants were positive about the possibility of completing the training on the web. The benefits of the web-based training that they mentioned were (1) doing it at your own pace; (2) easily accessible; (3) participate relatively anonymously; (4) participate from your own familiar environment; and (5) efficient, as there is no need to travel:

It was great that it was online so you can go through it at your own pace. It’s a much lower hurdle than if you have to go somewhere. If I had to go somewhere, I wouldn’t go.

You can go through it yourself, scroll through things that you find less interesting. Then you can go through it faster, at your own pace.

I’m not ashamed, but it might also be nice for other people that no one needs to know that you’re following this.

It’s nice that it’s not so personal. I don’t need to see these people in person. I can practice myself at home, so that’s nice.

Almost no disadvantages were mentioned regarding the web-based nature of the training. A few mentioned having to provide an email address as a disadvantage and the expectation that you would receive unwanted advertising as a result.

#### General Improvement Points

More strengths were mentioned than points for improvement. The user-friendliness of doing the SOC on the phone instead of on a laptop is important to some:

*On the phone it was difficult to open the program...There are some things in the program you really need to do on a laptop.* *It could have been a bit more phone friendly.*

Some of the respondents mentioned they preferred a less abrupt end of the program:

The closing was rather abrupt. Like: ready! But my problem was not solved yet. So, I would have liked some more follow-up steps: what can you do next? For example, a link to the SHC.

There was no conclusion or so, I thought. Of course, you could see how far you were, but it didn’t feel like an end.

Some respondents indicated that the design could be improved to make users stick around longer:

The website doesn’t look spectacular...I would prefer something more modern and more of this time.

#### Perceived Impact of the SOC

Although not all respondents thought that their complaints had been reduced by the SOC, all respondents indicated that they would recommend the SOC to other young people with similar problems or help requests. For most respondents, the following applies: if it does not help, it will not hurt either:

Whether or not it has added value will vary from person to person. But I think I would recommend it to others.

I didn’t benefit from it...But I’d say yes try it: there’s no harm in trying.

Almost all respondents indicated that they had learned something new through the SOC. Only 1 respondent, who had previously done a lot of research herself, stated that she had not learned anything new. The following gains were mentioned by multiple participants: they felt that they were normal and not the only one with the problem, they could communicate more and better with their partner about sex, their knowledge had increased, and they could reflect better:

I do have more insights. Especially the feeling that I’m not alone in this. At first, I thought, I must be the only one with this problem.

We started talking and thinking about it a lot more. It has become much more negotiable, much more concrete.

A number of respondents indicated that the SOC helped them seek further professional help:

So, it was a kind of push in the direction of the GP. SOC helped me identify what the pain felt like. The GP then referred me to a sexologist.

SOC did help me in seeking professional help. The training gave me courage to take the step towards a physiotherapist.

### Qualitative Evaluation by Nurses

#### Strengths

In general, the nurses were very positive about the SOC, both in terms of the content and format. Frequently mentioned positive aspects of the content were the high quality of the information (eg, clarity and reliability), quantity of the information (eg, not too much and not too little), and structure (eg, step by step). With regard to the format, the nurses appreciated that they could choose their own coach, the variety (eg, video, written text, library, exercises, and animations), and that participants could choose their own pace. Owing to the sensitivity of the subject, most nurses consider the digital form and the possibility of performing the exercises independently to be an important advantage for young people. They expected the SOC to have a low threshold:

Anonymous is nice and pleasant, that is accessible, they are still susceptible subjects.

A number of nurses indicate that at the same time, they find it a disadvantage that they do not see the clients back:

They liked being able to do the SOC digitally. They responded well to that. It’s hard not to see them again. But that is also a kind of advantage of SOC, that they can do it in anonymity. For some it is difficult to talk about it, then it is nice to get started with it yourself, safely.

#### Weaknesses

The biggest drawback that the nurses see in the SOC is that it demands a lot of time from young people and therefore may place too great a demand on them. This could be a reason for the participant dropping out. According to some nurses, young people often prefer quick solutions. Therefore, a number of nurses proposed to differentiate more. In addition to the SOC, they advise the development of a number of short videos in which the basics of common sexual problems (eg, premature ejaculation, sexual arousal, and pain during intercourse) are explained and to use the more extensive SOC only with more motivated clients:

And for example, a video about when sex hurts, that women are often not excited enough in that case. With short videos you can help more young people.

Not every nurse agreed with this advice because they believed that sexuality is broad and that different aspects should be addressed. That takes time. They recommend explicitly stating in the introduction that results depend on an investment of time:

The more time you spend on it, the more you get out of it. It is up to the young person themselves to decide how much time they want to put into it.

#### Perceived Additional Value of the SOC

Several nurses emphasize that the SOC is not a replacement for a consultation but an additional service that can be valuable. The SOC contains a lot of high-quality information according to the nurses, and there is not enough time to share it during consultation hours:

Sending a lot of information is not our job, we engage in motivational interviewing. So based on what the client contributes. We can, however, refer to SOC where there is more room for sending and information transfer.

Sometimes a young person only finds out after a consultation that pain during sex is not normal, so those young people will not look for a solution themselves because they do not know that it is not normal and that they can do something about it. You can refute that in a personal conversation, that is the power of a consultation.

Other nurses believe that it can replace a part of their tasks during consultation hours:

It can be a replacement for young people who do not come to the clinic. The group can then get to work. But it can also be a replacement for the consultation hour, if you don’t have time anymore...And that a lot has to be discussed in such a conversation. Then choose the most urgent question. So, it is better to immediately refer to SOC during a consultation hour and to schedule a follow-up consultation. Who knows, they have already started working themselves.

SOC can be a great addition, but also partly a replacement. Young people can look at this quietly and choose what they think is important. You don’t have that much time in a consultation hour.

#### Points for Attention for Future Use

A number of nurses consider it important to also use the SOC in the context of prevention. They found the basic module particularly suitable for this purpose.

Some nurses believe that a follow-up consultation or a phone call should be offered as a standard if a client is referred to the SOC by a nurse to see whether the problem has been resolved or whether further steps are desirable:

Making a circle: did it help you? What did it fix? How are you now? I would actually offer it as standard, the follow-up call. I think that’s neat. I also don’t think young people will call us any time soon if they still have something to worry about.

Some believe that a web-based follow-up consultation is suitable because it is also on the web:

It could be even easier with video consultations, then young people do not have to travel and it suits SOC because it is also online. So accessible and easy and it saves time. Follow-up physical consultation may be too much of a hassle.

Several nurses expect that it would be nice for young people who independently ended up at the SOC to be informed of the possibility of contacting the SHC or the helpline.

## Discussion

### Potential Reach of WSTPs

WSTPs attract a diverse group of users. Depending on the content of the program, both young men and women seemed equally interested in this type of web-based service. This is in line with international studies among middle-aged and older people, which show that help-seeking behaviors of men and women are quite similar [5,23].

Looking at the different age categories, the youngest group, aged 16 to 18, subscribes relatively less often to WSTPs. This may be explained by the difference in sexual activity between the age categories: 18 years is the age in the Netherlands at which (only) half of the young people have had sex [7]. Another sexually active group that is relatively underrepresented among the users of WSTPs is young people with a low educational level. In the Netherlands, health-seeking behavior on the internet depends on educational background. Highly educated people look for health information on the internet more often (84%) compared with those with intermediate (76%) or lower education levels (57%) [24]. In addition, digital skills vary depending on educational and cognitive levels. To achieve that WSTPs are maximally accessible to all target groups and prevent health inequities from worsening [9], the results of this study provide a clear indication that both when developing WSTPs and when promoting them, it is important to take into account the lower educated and digitally less skilled target groups.

The diversity among the subscribers is also reflected in the satisfaction with their sex life and the severity of the complaints they experienced. More than half of the subscribers (approximately 628/1028, 61.09%) were neutral or satisfied with their sex lives. Approximately one-eighth (126/1028, 12.26%) of the subscribers assessed the severity of complaints as low. On the basis of the interviews, it can be concluded that some of the subscribers were not seeking help at all; instead, they were just curious and created an account because of the possibility to participate for free. For users who experience mild complaints with only a limited impact on their satisfaction with their sex life, the easy accessibility of WSTPs enables them to work on their complaints at an early stage. From this perspective, WSTPs can be used in a preventive manner and can help prevent complaints from becoming more serious. In addition, it helps SHCs to focus on face-to-face consultations with clients expressing more severe needs.

In contrast, a substantial number of subscribers experiencing a high level of complaints (491/1028, 47.76%) have been dealing with them for >1 year (112/491, 22.8%) or 2 years (154/491, 31.4%) and are (very) dissatisfied (400/1028, 38.91%) with their sex life. The vast majority (921/1028, 89.59%) did not seek help from a professional for this in the past 2 years. These results confirm the findings of a British study [25] that WSTPs can meet the needs of groups of young people who experience serious complaints owing to sexual dysfunctions and are not reached by the health professionals. WSTPs can be less threatening and embarrassing than programs delivered in person [14,26].

This is also underlined by the routes taken by the subscribers to the SOC: social media appears to be the most important recruitment channel for the SOC (841/1028, 81.81%). As shown in a study in the United States, the internet is an important source of knowledge for men who seek information about premature ejaculation [27]. They concluded that the web-based information available to these men is often of uncertain quality and that there is a need for a reliable web-based offer by health professionals. The European Society for Sexual Medicine acknowledges that web-based treatment interventions can be effective but needs to be available to the public [26]. If WSTPs are offered without the guidance of a health professional, in addition to attention to the quality and reliability of the web-based treatment, it is also important to define the scope of the intervention. In the case of further implementation of the pilot WSTPs, it is important to make it clear at an early stage, for example, when creating an account, that it is not suitable for medical or physical complaints.

### Use and Drop Out of the WSTPs

This study shows that when WSTPs are offered free of charge and without involving a professional, a significant dropout (approximately 50%) should be expected, despite a relatively positive assessment of the form and content of the program. Such dropout rates (between 20% and 40%) are common in web-based health interventions because of the self-guided nature [3,26,28,29]. With regard to mental health, there is evidence for higher dropout in less educated participants and men [30]. The results of the interviews and FM with participants indicate that the dropout rate cannot be exclusively attributed to program characteristics or its perceived shortcomings. Making WSTPs available for free partly attracts people who want to try it out without obligation. In addition, complaints may be resolved before the program is completed, and part of the program may already offer sufficient support. Furthermore, a (sexual) relationship may have ended.

### Acceptance and Evaluation of WSTPs by Young People

In general, the participants were positive about the SOC. The important benefits of WSTPs highlighted in the international literature, as described in the *Introduction* section of this paper, are confirmed in this study. Valued aspects that emerge are doing it at your own pace; easily accessible; participating anonymously; participating from your own familiar environment; and efficient, as there is no need to travel to a professional. Unfortunately, owing to the relatively low response to the FM, no reliable statements could be made about the relationship between the assessment and background characteristics of the participants.

On the basis of the comparison of the BM and FM, it can be concluded that WSTPs can improve satisfaction with sex life and decrease complaints, at least among some users. This study shows that the positive assessment by young people does not depend exclusively on a positive result. The important elements of WSTPs, which are also appreciated by users who have not experienced improvements, are clear information and explanations and normalization of the problems. For some, knowing that they are not the only ones struggling with a particular sexual problem seems important in itself. For others, normalization lowers the threshold for communicating with their sex partner about their complaints or opens the way to seeking help from a health professional. From this perspective, WSTPs turn out to encourage young people to adopt solution-oriented behaviors.

At a practical level, several conditions emerge that increase the likelihood of a positive outcome. In addition to clear information and explanation, a variation in form (combination of video, written text, and practical exercises), a phased program build-up, and the possibility of choosing one’s own video coach contribute to the acceptance and appreciation of WSTPs. Offering the option to select more in-depth additional information and more practical and varied exercises increases the attractiveness of WSTPs. Concrete guidelines for the next steps after WSTPs and more actively motivating and remembering participants to complete the programs are warranted.

### Evaluation and Acceptance of the WSTP by Nurses

If the conditions of good quality and good connection with sexual health care professionals are met, nurses are generally of the opinion that WSTPs can be a good additional service to the regular consultation hours of the SHCs and can offer significant benefits to young people and health professionals. According to some nurses, WSTPs can be regarded as an extension of a regular consultation within which time is limited. Most of them emphasize that WSTPs are no replacement of regular consultations because a dialogue is missing and users are not offered the possibility to ask questions directly. These results confirm that acceptance of and resistance to new web-based technologies in health care depend on individual opinions of how they might (negatively) interfere with the ability to make good diagnoses or build relationships with clients [17].

According to nurses, an advantage of offering WSTPs is that they can increase the efficiency of the consultation hours. By letting clients get started with WSTPs in the first instance, they are given the opportunity to inform themselves and perform exercises that give them a better understanding of the problems they experience. If nurses offer a follow-up consultation, clients are likely to be better able to formulate their request for help.

An important point of attention for nurses is the extent to which youngsters are sufficiently stimulated to complete the training. Some nurses doubt whether WSTPs do not demand too much of young people’s time because of the wealth of information. They see opportunities for a version of the SOC with shorter videos for a less motivated target group. The results of the evaluation among young participants are not directly in line with this doubt. One of their regular-mentioned suggestions for improvement is the provision of more in-depth information. However, the limited participation of young people with a lower level of education in the postmeasurement and interview samples can cause a bias. It is recommended to actively involve more susceptible groups of young people in the (further) development of WSTPs to find the right balance in this matter.

### Limitations of This Study

Finally, despite the response-enhancing measures, too few users participated in the FM, which means that no reliable conclusions can be drawn regarding the efficacy of the SOC.

The implementation pilot provides a good estimate of the potential reach of WSTPs among young people. However, owing to the relatively high dropout and nonresponse in the FM and the relatively low number of interviews, a possible risk of bias should be noted with regard to findings concerning acceptance, assessment, and perceived efficacy of the WSTPs. A recent review study mentioned that for many internet- and mobile-based psychological interventions for sexual dysfunction, the risk of bias is high owing to high dropout [20]. Although participants who had completed the WSTPs as well as those who dropped out early were invited for the quantitative FM, it cannot be guaranteed that the results are representative of all participants who have started the WSTPs. From this perspective, the formulated conclusion of this pilot study should also be considered with some caution, and limiting nonresponse in future research is a major challenge.

Despite the incentives, the number of participants in the interviews in our pilot study was also disappointing (N=8). As a result, intended comparisons between subgroups have only been performed to a limited extent. For example, a comparison between differences in educational level or sexual preference is interesting.

The outcomes of the evaluation of the SOC among participants depended strongly on the quality of individual WSTPs. Although the various programs of the SOC have been positively assessed in advance by sexologists, they have not been pretested among young people. Pretest insights into (and adjustments based on) the strengths and weaknesses of the individual programs can not only be helpful in interpreting the results of this pilot in routine care but may also limit dropout.

Before young people could create an account, they had to complete the baseline questionnaire completely and provide an email address. This raises the threshold for young people to follow a training course at the SOC. This distorted the potential inflow.

Owing to the COVID-19 pandemic, the pilot has been postponed several times. The limitation of the opening hours of the SHCs and the long-term high workload it entailed have seriously hampered the implementation of the SOC. As a result, only a limited number of young people were referred to the SOC by a nurse, and very few follow-up consultations were offered. Therefore, this study can provide insufficient insight into the added value of the SOC in combination with the support of a nurse from the SHC, for example, whether according to the nurses, the SOC helps the client to further clarify the request for help or whether the nurse can work more efficiently because clients have prepared themselves through the SOC.

Simultaneously, it is clear that COVID-19 undermines the importance of easily accessible and effective WSTPs in several respects. Several review studies have shown that during the pandemic, sexual dysfunctions increase (more among women), and in COVID-19 long haulers, several complications related to erectile function arise [31-33]. Furthermore, it is argued that WSTPs could have an important role in allowing continued support at times of lockdown and preventing the worsening of sexual, mental, and physical health after the pandemic [31].

### Future Research

Given the important role that WSTPs can play in situations where face-to-face interventions are not available or are available to a lesser extent, it is important to pay more in-depth attention to dropout analysis, barriers, and facilitators of the acceptance of these web-based services in future research [29]. The results not only contribute to limiting dropout but also improve the acceptance of WSTPs. A review of the public acceptability of e-mental health treatment services shows that e-mental health treatment services in general are perceived as less helpful than traditional face-to-face interventions, and intentions for future use are smaller. In addition, it was concluded that therapist-assisted e-mental health services were preferred over unguided programs [34]. The question is to what extent is this also the case for a taboo subject such as sexual dysfunction? In this context, it is very interesting for future research to examine the possibilities of integrating artificial intelligence–based support into the WSTPs. Promotional strategies aimed at promoting public acceptance of artificial intelligence in health care processes seem to be effective [35].

### Conclusions

WSTPs can reach a large number of young people with sexual problems who are less likely to seek help from a health professional. By offering the WSTPs free of charge and with a low threshold (eg, via social media), it appears to be easy for young people to participate. This can potentially have a preventive effect, as young people with no or only mild complaints are encouraged to try out the WSTPs before complaints become more serious. Simultaneously, it can be concluded that getting in easily also means getting out quickly. This results in a relatively high dropout rate.

Participation in WSTPs can directly or indirectly benefit young people. Direct benefits occur for some of the participants and are expressed in a decrease in the level of complaints, higher satisfaction with sex life, and more knowledge and understanding of the complaints experienced. Indirect benefits relate to the normalization of sexual problems, making it easier to communicate with a sex partner, or seek further help from a health professional. Therefore, WSTPs should not be offered as stand-alone interventions or as a replacement for regular consultation hours but as an additional service and referring to them.

To achieve maximum access for all target groups and limit dropouts, people of all backgrounds, especially those with low educational level and limited digital skills, should be involved in designing and promoting the WSTPs. On a practical level, the following elements contribute to the acceptance and appreciation of WSTPs: clear information and explanations, a combination of video and text content, practical assignments, and the possibility of choosing between different video coaches. To meet the needs of different audiences, options for users to easily scale up to more in-depth information or more specific exercises increase the appeal and acceptance of WSTPs.

## Abbreviations

BM: baseline measurement
FM: follow-up measurement
SHC: sexual health clinic
SOC: Sense Online Coach
STI: sexually transmitted infection
WSTP: web-based self-training programs on sexual dysfunction

## Data Availability

The data sets generated and analyzed during this study are not publicly available because of the high sensitivity of the subject of the study but are available from the corresponding author upon reasonable request.
